# Cytotoxicity of Dendrimers

**DOI:** 10.3390/biom9080330

**Published:** 2019-08-01

**Authors:** Anna Janaszewska, Joanna Lazniewska, Przemysław Trzepiński, Monika Marcinkowska, Barbara Klajnert-Maculewicz

**Affiliations:** 1 Department of General Biophysics, Faculty of Biology and Environmental Protection, University of Lodz, 90-236 Lodz, Poland; 2 Leibniz Institute of Polymer Research Dresden, 01069 Dresden, Germany

**Keywords:** dendrimers, cytotoxicity, drug delivery, nanoparticles, macromolecules, toxicity

## Abstract

Drug delivery systems are molecular platforms in which an active compound is packed into or loaded on a biocompatible nanoparticle. Such a solution improves the activity of the applied drug or decreases its side effects. Dendrimers are promising molecular platforms for drug delivery due to their unique properties. These macromolecules are known for their defined size, shape, and molecular weight, as well as their monodispersity, the presence of the void space, tailorable structure, internalization by cells, selectivity toward cells and intracellular components, protection of guest molecules, and controllable release of the cargo. Dendrimers were tested as carriers of various molecules and, simultaneously, their toxicity was examined using different cell lines. It was discovered that, in general, dendrimer cytotoxicity depended on the generation, the number of surface groups, and the nature of terminal moieties (anionic, neutral, or cationic). Higher cytotoxicity occurred for higher-generation dendrimers and for dendrimers with positive charges on the surface. In order to decrease the cytotoxicity of dendrimers, scientists started to introduce different chemical modifications on the periphery of the nanomolecule. Dendrimers grafted with polyethylene glycol (PEG), acetyl groups, carbohydrates, and other moieties did not affect cell viability, or did so only slightly, while still maintaining other advantageous properties. Dendrimers clearly have great potential for wide utilization as drug and gene carriers. Moreover, some dendrimers have biological properties per se, being anti-fungal, anti-bacterial, or toxic to cancer cells without affecting normal cells. Therefore, intrinsic cytotoxicity is a comprehensive problem and should be considered individually depending on the potential destination of the nanoparticle.

## 1. Introduction

**Figure 1:**
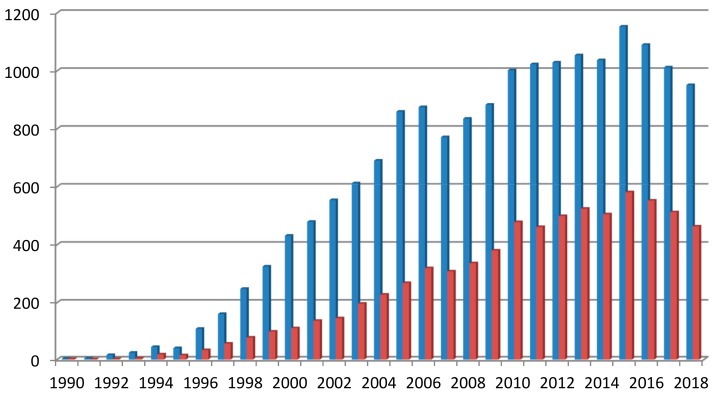
Number of articles about dendrimers in Scopus (blue bars—all fields; red bars—biomedical field).

Dendrimers, also known as starburst polymers [1], cascade molecules, or arborols [2], were developed in 1980s. Since the pioneering work of Tomalia [1], Newkome [2], and Vögtle [3], numerous research groups contributed to both the development of new dendrimer types, as well as their biomedical applications. Figure 1 illustrates the increasing number of biological and chemical publications on dendrimers in the last two decades. In the past five years, an average of 1000 publications per year appeared in the dendrimer field. This raises the question as to what makes dendrimers so interesting. There are many logical answers. 

**Figure 2:**
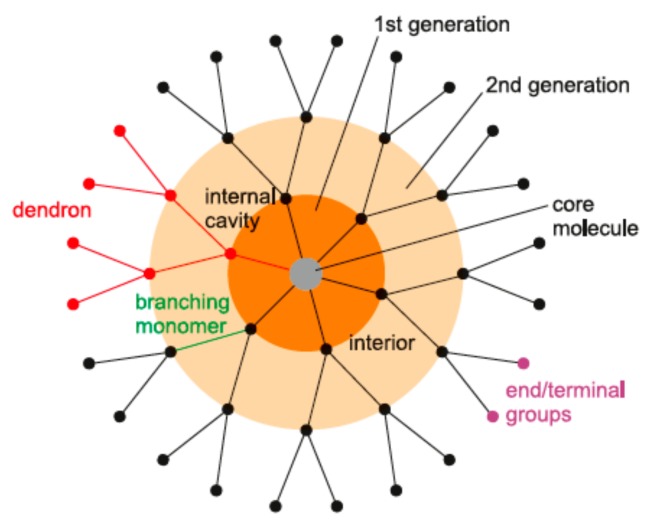
Structure of a dendrimer.

Firstly, dendrimers are characterized by a compact structure and a strictly predictable molecular weight (MW). They are monodisperse, polyvalent, usually globular macromolecules with a regular and highly branched three-dimensional architecture. They consist of a central core molecule surrounded by branches called dendrons, and are terminated with functional surface groups. The essential feature of these nanomolecules is their generation, defined as the number of layers attached to the core and denoted as Gn, where n can range from 0 to 12. Figure 2 illustrates the general structure of a dendrimer molecule. Secondly, it is possible to precisely design the structure of the dendrimer. During synthesis, one can control branching (topology) and introduce modifications at terminal end groups. Thirdly, most dendrimers are soluble in aqueous solutions, which is fundamental for drug candidates because it determines good absorption and bioavailability. Last but not least, many dendrimers easily penetrate cell membranes and increase cellular uptake of whatever is complexed by or conjugated with them [4]. 

**Figure 3:**
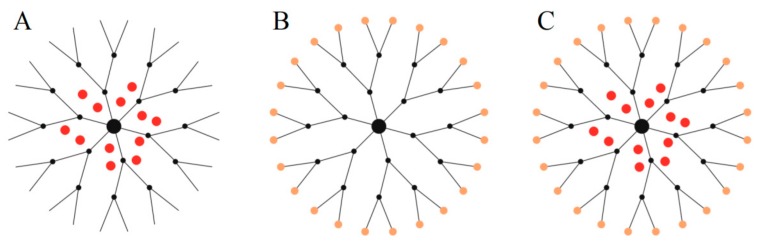
Three ways of complexation or conjugation of guest molecules with a dendrimer molecule: encapsulation in the internal cavities (**A**), attachment to the periphery (**B**), or both methods simultaneously (**C**).

Figure 3 shows three possible uses of dendrimers as nanocarriers. Thanks to their specific structure and the presence of controllable internal cavities, these nanomolecules are perfect for the encapsulation of guest drugs (Figure 3A). Moreover, bioactive compounds can be attached to reactive groups at the periphery of the dendrimer (Figure 3B). The same dendritic molecule can serve as a carrier of both encapsulated and surface-conjugated compounds (Figure 3C). Additionally, the periphery of the dendrimer can be easily modified to improve properties such as solubility or cytotoxicity [5].

All these properties make dendrimers excellent candidates for nanomedical applications. As compounds that are so intensively studied for their potential use in biomedicine, dendrimers should meet several criteria. Specifically, they should be non-toxic, non-immunogenic, biopermeable (possess the ability to cross biobarriers), able to stay in blood circulation until the desired effects occur, and able to target specific biological structures. Many groups of dendrimers fulfill most of these requirements; however, their utilization in biomedicine is often limited due to their high cytotoxicity [6]. This review initially presents the most researched potential applications of dendrimers in nanomedicine. Then, it discusses important aspects of dendrimer cytotoxicity. Next, we provide insight into the problem of cytotoxicity of cascade molecules in the aspect of their clinical application. Finally, we discuss whether the toxicity of dendrimers limits their biomedical applications, and what the main strategies to improve their biocompatibility are.

## 2. Biomedical Potential and Applications of Dendrimers

**Figure 4:**
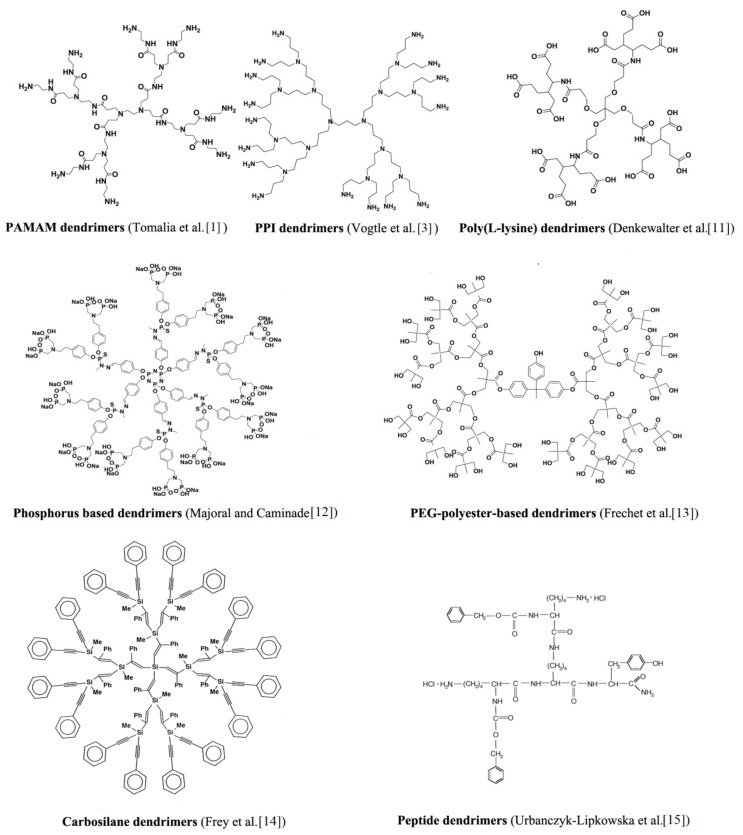
Chemical structures of selected types of dendrimers.

Poly(amido amine) (PAMAM) and poly(propylene imine) (PPI) dendrimers are the two most commonly studied dendrimers, probably due to their broad commercial availability [7]. However, phosphorous dendrimers (P-dendrimers) [8], carbosilane dendrimers (CBS), poly(L-lysine) dendrimers (PLL), polyesters (PGLSA-OH) [9], poly(2,2-bis (hydroxymethyl)propionic acid dendrimers (bis-MPA) [10], and peptide dendrimers are also extensively explored. Figure 4 shows structures of selected types of dendrimers [1,3,11,12,13,14,15]. All these different groups of dendrimers were investigated for their use in biomedical applications as nanodrugs, drug delivery systems, contrast agents for magnetic resonance imaging (MRI), genetic material carriers, nanoscale containers, artificial proteins, synthetic vaccinations, antiviral and antibacterial agents, or therapeutic factors in neurodegenerative disorders. Several examples are presented below.

Boas et al. showed that thiourea-functionalized PPI dendrimers can be used as a drug delivery system. The encapsulation properties of these dendrimers are based on the formation of dense, hydrogen-bonded surface shells with solid-state characteristics. Small guest molecules captured in the interiors of the dendrimers were unable to escape even after extensive dialysis. The number of encapsulated guest molecules was directly proportional to their size and shape. Such a dendrimer box could be opened controllably by partial or total hydrolysis to release either some or all of the entrapped guest molecules [16]. In addition, PAMAM dendrimers of generations 3 and 4 were used as nanoscale containers for the anti-inflammatory drug ibuprofen. Up to 78 ibuprofen molecules were incorporated into one molecule of a PAMAM-G4-NH_2_ dendrimer through electrostatic interactions between the dendrimer amines and the carboxyl group of the drug. The complexed drug entered A549 cells much more rapidly than the pure drug, suggesting that dendrimers were able to carry the complexed drug inside cells efficiently [17]. Another drug, cisplatin, commonly used in cancer treatment, was encapsulated in PAMAM dendrimers [18]. Interestingly, in comparison to free medicament, the complex showed slower drug release, higher accumulation in solid tumors, and lower cytotoxicity, and could be effective even on cisplatin-resistant cells [19,20]. Khandare et al. presented the current status and perspective of dendritic polymer nanoconjugate platforms (e.g., PAMAM dendrimers and dendritic polyglycerols) for cellular localization and targeting of specific tissues [21], while Gupta et al. highlighted in their review the synthetic progression and biomedical applications of non-ionic polyether-based amphiphilic architectures as delivery systems of active components [22]. Photodynamic therapy (PDT) is a promising light-based treatment method, utilizing compounds called photosensitizers for selective damage of diseased cells and tissues. The photosensitizer molecule, when activated by light, induces the formation of reactive oxygen species (ROS), which are toxic to target cells. However, common problems limiting the application of photosensitizers regardless of their excellent ROS generation capability are their intrinsic cytotoxicity and insufficient lipophilicity. Therefore, dendrimers can be employed to improve the efficacy of these compounds. For example, PAMAM dendrimers were used for the delivery of the photosensitizer rose bengal (RB) to Dalton’s lymphoma ascite (DLA) cells—a cancer cell line model. The dendrimer–RB formulation showed high phototoxic efficiency toward the tested cell line and the dendrimer-based delivery system reduced the dark toxicity of RB [23].

Dendrimers can be used as therapeutic factors in neurodegenerative disorders. In vitro studies showed that dendrimers were capable of interfering with the formation of amyloid fibrillar structures typical to the onset and development of neurodegenerative disorders, such as Alzheimer’s, Parkinson’s, and prion diseases. Wasiak et al. demonstrated that cationic phosphorus dendrimers of generations 3 and 4 (CPD G3, CPD G4) were able to affect β-amyloid and MAP-Tau protein aggregation processes. Assessment of the cytotoxicity of formed fibrils and intermediate products during Aβ 1–28 aggregation was performed using a neuroblastoma cell line (N2a). The results indicated that CPDs were able to reduce the toxicity of aggregated forms of Aβ 1–28 [24]. Similarly, the GATG (gallic acid–triethylene glycol) dendrimer decorated with 27 terminal morpholine groups ([G3]-Mor) [25] and glycodendrimers, such as maltose-decorated PPI dendrimers [26], were able to interfere with the aggregation process of Alzheimer’s Aβ peptides. Moreover, dendrimers seem to be a useful tool for therapy of synucleinopathies. This group of neurodegenerative pathologies, including Parkinson’s disease and dementia with Levy bodies, is caused by the intracellular aggregation of insoluble protein α-synuclein in neural tissue. On this note, α-synuclein clearance in vitro was reported for viologen–phosphorus dendrimers [27], urea- and thiourea-modified PPI [28], and CPDs [29]. CPDs also exhibited activity against fibrillation and aggregation of prion peptides [30].

Dendrimers and dendrimer complexes proved to be effective antipathogenic agents in vitro. Unmodified and maltose-modified PPI dendrimers were tested for their potential against a set of pathogenic bacteria and yeast—*Candida albicans*. It was shown that maltose-modified PPI dendrimers displayed the highest antibacterial activity and a striking selectivity toward a Gram-positive bacterium, *Staphylococcus aureus*. Simultaneously, these nanomolecules exhibited insignificant toxicity toward eukaryotic cells [31]. Moreover, the encapsulation of silver salts in PAMAM dendrimers increased antimicrobial activity against various Gram-positive bacteria in comparison to unconjugated silver salts [32]. These conjugates were shown to be effective for antimicrobial preservation and the protection of textiles [33,34]. PAMAM dendrimers were also demonstrated to have potential as topical microbicides against herpes simplex virus (HSV) infections. All of the tested PAMAM compounds were active against both virus types (types 1 and 2) in the cytopathic effect (CPE) inhibition assay, in which the drug was added to cells prior to the addition of the virus [35]. One of the most advanced studies on dendritic nanomolecules as antiviral agents concerns PLL dendrimers modified with sulfonated naphthyl groups. They were extensively studied as a topical microbiocide for sexually transmitted infections (STIs). The nanodrug exhibited activity against human immunodeficiency virus (HIV), HSV, and bacterial vaginosis [36]. The PLL-based product, called VivaGel^®^, was developed by Starpharma (Melbourne, Australia). Preclinical development studies demonstrated that VivaGel^®^ was 100% effective at preventing infections of primates exposed to a humanized strain of simian immunodeficiency virus (SHIV). In 2012, the company started crucial phase III clinical trials for the treatment of bacterial vaginosis. First, VivaGel^®^-containing condoms were approved on the Australian market. Then, the product was approved for the treatment and prevention of bacterial vaginosis (BV) and the prevention of sexually transmitted infections [37].

Many researchers aimed to use dendrimers for vaccine and immunization purposes. A multiple antigenic peptide (MAP) system, based on PLL dendrimers, was pioneered by Tam et al. in the late 1980s [38,39]. It can be synthesized with defined mixtures of B- and T-cell epitopes. A MAP construct, modified with *Plasmodium falciparum* T- and B-cell stimulatory peptides, is a good example of such a system [40]. Furthermore, it was demonstrated using cancer-related peptides that MAPs were processed in antigen-presenting cells in the same way as antigens derived from intracellular pathogens (e.g., viruses), thereby providing a powerful immune response, including cytotoxic T-cells [41].

Since the pioneering work of Lauterbur, Wiener, and Tomalia et al. [42], numerous research groups contributed to the development of dendrimer-based MRI contrast agents. Longmire et al. in their reviews presented a broad insight into physicochemical properties, clinical applications, and in vivo results of nanosized MRI contrast agents with dendrimer cores. Generally, these contrast agents are considered to have enhanced r1 relaxivity compared to traditionally used low-molecular-weight metal chelates [43]. Moreover, due to multivalency, dendrimers have tunable pharmacokinetics which can be adapted specifically to imaged organs.

Many studies focused on the utilization of amino-terminated PAMAM or PPI dendrimers as non-viral gene transfer agents, increasing the transfection efficiency [44,45]. Dendrimers are able to bind and protect DNA, and they can be also used as a targeting factor in gene therapy. For example, Wong et al. constructed PAMAM dendrimer polyplexes conjugated with DNA and ligands such as folate or riboflavin obtaining multivalent vectors for targeted gene delivery [46]. Also, dendritic poly(L-lysines) (DGLs) deliver the small interfering RNA (siRNA) oligonucleotide with the same efficiency as Lipofectamine 2000. Furthermore, DGL had very high binding affinity to DNA, protecting it against DNAse I attack and simultaneously internalizing it into cells [47].

Interestingly, dendrimers were also investigated for their use in biomedical applications as artificial proteins. Owing to their nanoscale dimensions, globular shape, and other properties, they can mimic many important proteins. For example, the size of insulin (3 nm) is comparable to the size of a PAMAM G3 dendrimer. Similarly, cytochrome C (diameter = 4 nm) and hemoglobin (diameter = 5.5 nm) correspond to the sizes of PAMAM G4 and G5, respectively. A comprehensive strategy for designing dendritic nanostructures to mimic globular proteins was described by Tomalia et. al. [48]. They synthesized PAMAM dendrimers with a disulfide moiety in their core. Therefore, their shape and size can be precisely controlled with the application of traditional redox chemistry, allowing the synthesis of structures similar to naturally occurring peptides. Moreover, the dimensions of dendrimers can be compared to other biological structures. For instance, generations 5 and 6 of PAMAM dendrimers possess diameters approximately equivalent to the thickness of lipid bilayer membranes of biological cells, while generation 2 PAMAM dendrimers correspond to the width of DNA duplexes [44]. 

Dendrimers possess great biomedical potential and, as presented in Figure 1, new kinds of dendrimers and modifications of already existing ones are constantly being developed. Knowledge of the cytotoxicity of dendrimers is highly significant in the context of developing effective and safe dendrimer-based nanoparticles for biomedicine. The following sections provide a compact summary of the information on the cytotoxicity of several groups of dendrimers. The comparison of dendrimers and their behavior toward various cell lines will help to indicate the direction of further improvements of dendrimers and to distinguish the most promising candidates for future nanomedical applications.

## 3. Cytotoxicity of Dendrimers

### 3.1. General Aspects

**Figure 5:**
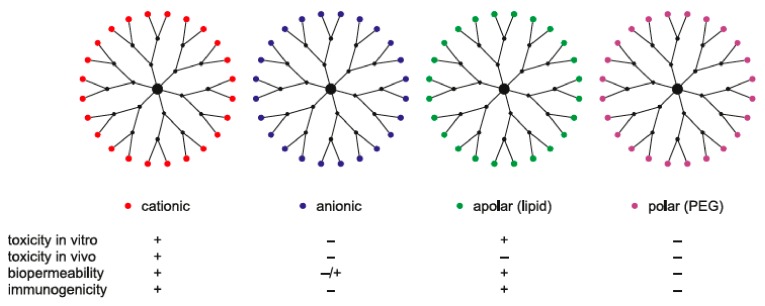
Influence of the surface charge of dendrimers on their bioavailability, immunogenicity, and their in vitro and in vivo toxicity. “+” indicates that there is an effect; “−” indicates no effect.

The cytotoxicity of dendrimers depends strongly on the number and nature of functional surface groups. Cationic dendrimers often exhibit high toxicity, whereas anionic and neutral dendrimers show slight or no toxic effects. For example, poly(amido amine) (PAMAM) and poly(propylene imine) (PPI) dendrimers possessing terminal primary amines are characterized by concentration- and generation-dependent toxicity [49,50], whereas grafted carbosilane–poly(ethylene oxide) (CSi–PEO) dendrimers and other dendrimers terminated with neutral or anionic groups seem to be much less toxic [51]. Thus, modification of the surface of the cationic dendrimer with negatively charged or neutral moieties decreases its cytotoxicity [50]. Surface functionalization with polyethylene glycol (PEG), pyrrolidone, or another biocompatible compound can significantly reduce cytotoxicity to levels far better than those of currently available products [51]. The cytotoxicity of cationic dendrimers can be explained by the interaction between negatively charged cell membranes and the positively charged dendrimer surface. This interaction leads to the formation of nanopores in the cell membrane, its damage, subsequent leakage of cellular content, and eventually cell death. Figure 5 illustrates how the surface charge affects the bioavailability, immunogenicity, and the in vitro and in vivo toxicity of dendrimers.

Mukherjee et al. published an interesting paper, disclosing an indirect impact of PAMAM dendrimers on cell viability. The authors observed generation-dependent toxicity of PAMAM G4, G5, and G6 to human keratinocytes (HaCaT) and primary colon adenocarcinoma cells (SW480). Based on spectroscopic methods, and the measurements of the zeta size and potential of dendrimers in fetal bovine serum (FBS)-supplemented medium, it was found that dendrimers absorbed proteins from the culture media. These data suggested the indirect mechanism of PAMAM cytotoxicity resulting from the depletion of medium components [52]. In the next paper, a direct influence of PAMAM G4–G6 on both cell lines was revealed. Specifically, the generation-dependent cytotoxicity correlated with the formation of ROS, increased lysosomal activity, induction of apoptosis, and DNA damage. These effects were consistent with a pathway of localization of PAMAM dendrimers in the mitochondria. ROS production was co-located in the mitochondria, and both generated levels and timescales were systematically generation dependent (G4 < G5 < G6) [53]. Mukherjee and Byrne also showed that early-stage HaCaT cell responses to PAMAM G4–G6 were associated with endosomal encapsulation, while later-stage responses were associated with mitochondrial attack. In all cases, the magnitude and evolution of responses depended on the dendrimer generation and dose [54]. Generation-dependent cytotoxicity was also observed for photosynthetic microorganisms, i.e., green algae and cyanobacteria. Expectedly, PAMAM-G4 dendrimers inhibited the growth of the microbes significantly more than G3 and G2. However, ROS formation was found only for OH-terminated PAMAM dendrimers. It was strictly mitochondria-related, and neither chloroplasts nor photosynthetic membranes were affected [55]. PAMAM dendrimers of generations G4, G5, and G6 were also evaluated for their aquatic toxicity using the test models *Vibrio fischeri, Daphnia magna, Thamnocephalus platyurus*, and two fish cell lines. The toxicological response correlated well with the dendrimer generation and, therefore, with the particle surface area; an increase in surface area led to an increased toxic response. *Daphnia magna* was found to be the most sensitive model, and the RTG-2 fish cell line was the least sensitive [56]. Interestingly, in vivo research utilizing an embryonic zebrafish model showed that the dendrimer surface is a more significant indicator of dendrimer cytotoxicity than generation and class [57]. 

Several types of dendrimers (PAMAM, PPI with either a diaminobutane (DAB) or diaminoethane (DAE) core, poly(ethylene oxide) (PEO)–grafted carbosilane (CSi–PEO), and polyether dendrimers) were compared regarding the influence of generation (G1–G4, G1.5–9.5), and surface functionality (–NH_2_, COONa, COOH, PEO) on their in vitro behavior. Dendrimers with amino terminal groups displayed concentration- and generation-dependent hemolysis, while anionic and CSi–PEO dendrimers were neither hemolytic nor cytotoxic toward a panel of cell lines in vitro. Polyether dendrimers with carboxylate and malonate surfaces were not hemolytic at 1 h; however, after 24 h, unlike anionic PAMAM dendrimers, they were lytic [50]. Kuo et al. analyzed cell death processes (apoptosis and necrosis) after the treatment of RAW 264.7 murine macrophage-like cells with PAMAM and PPI–DAB dendrimers. Exposure of cells to both types of cationic dendrimers led to a typical dose-dependent cytotoxicity. Assessment of cell morphology, the presence of a DNA ladder in gel electrophoresis, and cell-cycle studies indicated the induction of apoptosis. The authors also compared the sensitivity of different cell lines and found that tested dendrimers did not induce apoptosis in mouse fibroblast (NIH/3T3) cells and mouse liver (BNL CL.2) cells, in contrast to RAW 264.7 cells [58].

Since PAMAM and PPI are the most extensively researched among dendrimers, a very broad range of applications were proposed for these systems. A comprehensive review of studies aiming to reduce PAMAM and PPI cytotoxicity is presented below along with studies of arborols. 

### 3.2. Reduction of PAMAM and PPI Dendrimer Cytotoxicity

As mentioned above, surface modification is a common way to increase the biocompatibility of dendrimers. For example, modified PAMAM dendrimers of generation G4 with 4-carbomethoxypyrrolidone surface groups (PAMAM–pyrrolidone dendrimer) showed only minor toxicity and no ability to induce apoptosis. The most important finding was the lack of influence of the PAMAM–pyrrolidone dendrimer on intracellular ROS level and mitochondrial membrane potential even at the highest tested concentration (200 µM) [59,60]. PAMAM cytotoxicity can also be reduced by more comprehensive structural modifications. The toxicity of carboxymethylchitosan/poly(amidoamine) (CMCht/PAMAM) dendrimers toward the glioblastoma GBM cell line (U87MG) and human immortalized astrocytes (hTERT/E6/E7) was investigated. Short-term (1, 6, 12, 24, and 48 h) exposures to doses of 200 and 400 μg/mL did not cause a decrease in the cell viability, and long-term exposures (seven days) to CMCht/PAMAM induced only slight cytotoxicity in both cell lines (20% decrease in metabolic activity). Moreover, after a 48-h treatment, both cell lines presented 100% dendrimer internalization efficiency for the tested concentrations. These results suggest that CMCht/PAMAM nanoparticles may be an attractive drug delivery system for brain tumor treatment [61]. An interesting group of dendrimer-based compounds are tecto-dendrimers. They can be defined as polymers of higher architectural order, which are composed of a central dendrimer molecule and many dendrimers attached to its periphery. They are synthesized by a controlled introduction of covalent linkages between dendrimer building blocks. Schilrreff et al. synthesized saturated shell core–shell tecto-dendrimers using amine-terminated PAMAM G5 as a core and carboxyl-terminated PAMAM G2.5 as a shell (G5G2.5 tecto-dendrimers). The toxicity of this construct was then examined on epithelial cells. Preliminary results suggested that concentrations of G5G2.5 that did not damage healthy keratinocytes (HaCaT cell line) and showed antimelanoma activity (SK-Mel-28 cell line) [62]. Another surface modification of PAMAM dendrimers (G3 and G4) was based on the attachment of *N*-(2-hydroxydodecyl) groups. Dose–response effects of both modified and unmodified PAMAM were examined on two cell lines—a fish cell line (RTG-2) and a rat hepatoma cell line (H4IIE). No toxic effects of amino-terminated PAMAM dendrimers were observed for both H4IIE and RTG-2 cells lines when the concentration was below 500 μg/mL. For surface-modified PAMAM dendrimers, higher cytotoxicity was found for the H4IIE cell line [63]. Wen et al. revealed that the cytotoxicity of unmodified PAMAM (PG4) and histidylated PAMAM (HPG4) was a function of the polymer concentration and polymer/DNA ratio. It increased with an increased N/P ratio, but the cell viability after HPG4 treatment was significantly higher compared to that of PG4 at all investigated levels. The introduced histidine moieties might be one of the factors that reduced the toxicity of PAMAM, as protonated imidazole rings are regarded to be less cytotoxic than protonated amine groups. Moreover, HPG4/pDNA compared to PG4/pDNA showed improvements on cellular uptake, serum tolerance, cytotoxicity profile, and endosomal escape [64]. In another work, Ciolkowski et al. demonstrated how the reaction of PAMAM G4 with dimethyl itaconate resulted in the transformation of surface amine groups into pyrrolidone derivatives and, at the same time, reduced dendrimer toxicity. Modified PAMAM G4 did not affect mouse neuroblastoma cell line viability and showed no hemolytic activity [51]. The work of Jevprasesphant et al. confirmed that properties of PAMAM dendrimers can be significantly changed by surface engineering. Their permeation properties and toxicity to the colon adenocarcinoma cell line (Caco-2) increased with both concentration and generation and they were greater for cationic dendrimers (G2, G3, G4) than for anionic ones (G2.5, G3.5). The cytotoxicity of positively charged nanomolecules was reduced by conjugation with lauroyl chloride; the least cytotoxic conjugates were those with six attached lauroyl chains. Moreover, such a modification of cationic PAMAM dendrimers also increased their permeation through Caco-2 cell monolayers. Both PAMAM dendrimers and lauroyl–PAMAM dendrimer conjugates were able to cross epithelial monolayers via paracellular and transcellular pathways [65]. In their next work, Jevprasesphant et al. showed the cytotoxicity of PAMAM (G2, G3, G4) conjugated either with lauroyl chains or PEG 2000. The toxicity of both PEGylated and lauroyl-modified dendrimers toward Caco-2 cells was appreciably lower than that of the cationic counterparts (whole generations); four PEGs or six lauroyl chains were particularly effective in decreasing cytotoxicity [49]. Janaszewska et al. tested acid-terminated PAMAM G3.5 and amino-terminated PAMAM G4 in comparison to unmodified amino-terminated PPI-G4 and maltotriose-modified PPI-G4 dendrimers. Cationic PPI-G4 and PAMAM G4 were the most harmful for both Chinese hamster ovary (CHO) and human ovarian carcinoma (SKOV3) cell lines, especially in high doses. A maltotriose modification significantly reduced toxicity for the series of PPI-G4 dendrimers. A SKOV3 cell line, moderately resistant to doxorubicin and cisplatin, was more vulnerable to modified PPI dendrimers than the CHO cell line, which did not show resistance to the majority of anticancer agents. These findings imply that maltotriose-modified PPI-G4 dendrimers might be potentially interesting for anticancer therapy [66]. In another work, PPI G5 dendrimers were functionalized using protected glycine and phenylalanine, mannose, and lactose. Again, these dendrimers demonstrated a positive charge-based time- and concentration-dependent toxicity to tested cell lines (transformed African green monkey kidney cell line (COS-7) and HepG2 cell line). Surface-modified nanomolecules exhibited an improved toxicity profile in comparison to the parent dendrimer [67]. It seems that the conjugation of amino acids and saccharides is an effective way of reducing PPI cytotoxicity, whereas the reduction of PAMAM cytotoxicity is based mostly on the neutralization of their positive charge on the surface. 

The ability of dendrimers to cross cell membranes is of much interest for their application in drug and gene delivery. Indeed, a safe alternative to the viral system used in gene therapy is a nonviral carrier, and dendrimers seem to be excellent for this role. Albertazzi et al. revealed that PAMAM dendrimers G2, G4, G6, and lipidated G4 possess similar properties to widely used cell-penetrating peptides (the arginine-rich motif derived from the HIV protein TAT). Furthermore, they are not toxic toward the HeLa cell line (1.5 μM concentration for 1 h) and, thanks to their chemical tunability, they represent an attractive option for drug and gene delivery [68]. Also, Choy et al. indicated that dendrimers, namely, PAMAM and polyethylenimine (PEI), are among the most promising gene-carrier candidates for efficient nonviral gene delivery. Unfortunately, these dendrimers induced both a low level of apoptosis and a high level of necrosis, as well as a moderate genotoxic effect [69]. However, Jafari et al. partially overcame the problem of toxicity using PAMAM G5 conjugated with PEG 3500, which turned out to be less toxic and to have a higher transfection rate in vitro than the original dendrimer [70]. Proper surface engineering can also be applied to control the internalization pathway of dendrimers. Vidal et al. used four PAMAM-G4 dendrimers with different surface compositions with a hippocampal cell line: unmodified, conjugated with PEG on 30% and 50% of the surface, and conjugated with folic acid (FA) on 25% of the surface. The clathrin-dependent pathway proved to be a basic one for PAMAM internalization. However, the dendrimer modified with FA was internalized with clathrin- and caveolae-mediated endocytosis [71]. 

Navath et al. demonstrated the development of biocompatible PAMAM G4 dendrimers, enabling the attachment of drugs (indomethacin and dexamethasone) and imaging agents (dexamethasone and FITC) without using any specific linkers. This could be achieved thanks to multiple amino-acid-based orthogonal surface groups at the dendrimer periphery. Furthermore, one of the two functional handles at the periphery was used to develop in situ forming hydrogels, whereas the other handle could be used for conjugating drugs. An in vitro cytotoxicity test and a hemolysis assay showed that the heterobifunctional dendrimers were non-cytotoxic in the 100 ng/mL to 1 mg/mL concentration range [72]. 

Krishna et al. reported the synthesis of several PPI dendrons and dendrimers, which were constructed by involving an ether as the linker component and an imine as a branching component. The adopted synthetic sequence allowed an alcohol, an amine, or a carboxylic acid to be installed at the peripheries. The carboxylic-acid-terminated dendrons and dendrimers were evaluated for their cytotoxic properties, and, while most dendrons and dendrimers did not exhibit any measurable cytotoxicity, even up to 100 μg/mL, the second-generation dendrimer with the benzenoid core exhibited mild toxicity at concentrations above 30 μg/mL [73]. It was reported that preventing electrostatic interactions of dendrimers with cellular membranes was a necessary step toward minimizing the toxicity of dendrimer-based delivery vehicles to the endothelium. This conclusion was based on the studies with PPI dendrimer conjugates showing cytotoxicity and time-dependent membrane disruption in cultured human umbilical vein endothelial cells (HUVEC). Chemical modification of the surface amines of the parental dendrimer to neutral acetamide or PEG functionalities eliminated their acute cytotoxicity. Cationic primary-amine-containing dendrimers demonstrated drastic, time-dependent changes in plasma membrane permeability and prominent cytotoxicity, while complete removal of the primary amines or masking of the cationic surface via PEGylation decreased the toxic effects [74]. 

### 3.3. Cytotoxicity of Other Kinds of Dendrimers

Wang et al. proposed the synthesis of a star polymer composed of amphiphilic block copolymer arms. The core of the star polymer was a PAMAM dendrimer, the inner block of the arm was lipophilic poly(ε-caprolactone) (PCL), and the outer block of the arm was hydrophilic PEG. The star-PCL polymer was synthesized first via ring-opening polymerization of ε-caprolactone with a PAMAM-OH dendrimer as an initiator. The PEG polymer was then attached to the PCL terminus via an ester-forming reaction. It is known that hydrophobic dyes and drugs can be encapsulated in the micelles. A similar effect was observed for the star polymer. A loading capacity of up to 22% (*w*/*w*) was achieved with etoposide, a hydrophobic anticancer drug. A cytotoxicity assay demonstrated that the star-PCL–PEG copolymer was non-toxic for cells, indicating that this type of block copolymer could be used as a drug delivery carrier [75].

Lazniewska et al. studied toxic responses of two low generations of cationic phosphorus dendrimers (CPDs) of generation 2 and generation 3 toward murine embryonic hippocampal cells (mHippoE-18) and N2a cells. The obtained results showed that CPDs G3 were highly cytotoxic at concentrations above 1 μM and at 0.7 μM for mHippoE-18 cells. A significant decrease in cell viability corresponded to severe changes in cellular processes, such as massive ROS generation. Unlike other cytotoxic dendrimers, which activated the apoptotic pathway in cells, CPDs caused a breakdown of cellular processes followed by a necrotic cell death [76]. The cytotoxicity and genotoxicity of CPDs G3 and G4 were also studied in human mononuclear blood cells, A549 human cancer cells, and human gingival fibroblasts (HGFs). These dendrimers at concentrations up to 10 μM induced a concentration-dependent decrease in cell viability. An apoptosis/necrosis assay revealed two main fractions of cells—viable and necrotic. Moreover, neither compound induced breaks in isolated DNA, and both could form complexes with this nucleic acid and condense it. Additionally, CPDs induced DNA cross-links in cells, as examined by a comet assay. Overall, the results suggest that CPDs G3 and G4 were cytotoxic and genotoxic for chosen human cells [77]. The cytotoxicity of fluorescently-labeled CPD G2, possessing a fluorophore (maleimide-type) linked to the core, was studied by Kazmierczak-Baranska et al., who showed that cytotoxicity of this compound was relatively low toward HeLa cell (up to 20 μg/mL) and A549 (up to 1 μg/mL), and even less toxic after 48 h than after 24 h, which, in comparison with the results of previous authors, confirms the generation dependency of the cytotoxicity of CPDs [78]. Novel multivalent copper(II)-conjugated P-dendrimers and their corresponding mononuclear copper(II) complexes were synthesized, characterized, and screened for antiproliferative activity against human cancer cell lines: human colon cancer (HCT116), hormone-responsive breast cancer (MCF7), ovarian carcinoma (OVCAR8), and human glioblastoma–astrocytoma, epithelial-like (U87), and two non-cancer cell lines, namely, proliferative human lung fibroblasts (MCR5) and quiescent endothelial progenitor cells, *Cyprinus carpio* (EPC) [78]. It was also shown that copper(II)-conjugated P-dendrimer cytotoxicity increased with the number of terminal moieties available and was boosted by the presence of complexed Cu atoms. Two dendrimers (1G3 and 1G3-Cu) were selected for antiproliferative studies against a panel of tumor cell lines and were demonstrated to have potent antiproliferative activities with IC_50_ values ranging from 0.3 to 1.6 μM. Interestingly, the complexation of the terminal ligands of 1G3 dendrimers by copper(II) metal strongly increased IC_50_ values in non-cancer cell lines [79].

Zeng et al. presented hyperbranched polyester Boltorn decorated with PEG groups, which showed potential as an efficient and specific delivery platform for drugs and imaging agents. The cytotoxicity, cellular uptake profiles, and intracellular trafficking of polymer micelles were tested in MDA-MB468 breast cancer cells. The uptake of these nanoparticles was positively correlated with time and concentration and was energy dependent. These nanoparticles were shown to be internalized into cells via clathrin- and macropinocytosis-mediated endocytosis. Moreover, they did not significantly affect the viability of the cells [80]. Hydroxylated polyester dendrimers from first to fifth generation were also used as stabilizing agents of silver particles. The antibacterial properties of the dendrimer-stabilized silver particles were tested against *Escherichia coli*, and the toxicity against human cells was evaluated with the human epithelial cell line A549. The silver particles, especially those prepared from dendrimers of higher generations, demonstrated a significant antibacterial effect against *E. coli*. No toxicity against human cells was observed for the silver particles even in case of the highest investigated silver concentration. Therefore, such a construct could offer great potential for application as an antibacterial agent with low human toxicity [81]. Feliu et al. also showed that bis-MPA aliphatic polyester dendrimers were degradable and non-cytotoxic to human cell lines and primary cells. Two different chemical surfaces (neutral with a hydroxyl end group and anionic with a carboxylic end group) and dendrons corresponding to the structural fragments of the dendrimers were examined. Cell viability studies were conducted in human cervical cancer (HeLa) and acute monocytic leukemia cells (THP.1) differentiated into macrophage-like cells, as well as in primary human monocyte-derived macrophages. The authors observed excellent biocompatibility for the entire hydroxyl functional bis-MPA dendrimer library [82]. The biological evaluation of a library of eight polyester dendrimer–poly(ethylene oxide) (PEO) bow-tie hybrids was also performed. The library included polymers characterized by MWs from 20,000 to 160,000 and architectures with the number of PEO arms ranging from two to eight. In vitro experiments revealed that the polymers were non-toxic to cells and were degraded to lower MW species at pH 7.4 and pH 5.0 [83]. 

One more architecture characterized by improved biological properties is dendritic polyglycerol (dPG). Various dPG derivatives possessing neutral, cationic, and anionic charges were compared with amine- and hydroxyl-terminated PAMAM dendrimers. In the U-937 cell line, dPG with terminal hydroxyl groups, dPG sodium sulfate, dextran, and linear PEG caused no toxic effect compared to dexamethasone used as a reference standard. Similarly, amine terminal dPGs (7% and 18% amines) were also almost non-toxic (cell viability equal to 90%). On the other hand, three polymers, namely, dPG with higher amine functionality (45% and 100%), PAMAM G4 hydroxyl and amine dendrimer, and PEI, caused higher cell toxicity than the uncharged hydroxyl dPGs, dextran, and PEG. The cell compatibility results showed that the dendritic polyglycerols were as safe as linear PEG polymer or dextran, which indicates the suitability of dPG derivatives in delivering therapeutic agents [84].

Fuchs et al. developed a new series of dendrimers G1 and G2 possessing various surface functionalities. Terminal amine groups of these dendrimers were decorated with protons, *tert*-butoxycarbonyl (Boc) or benzyloxycarbonyl (Cbz) protecting groups, Boc-protected or unprotected natural amino acid residues, ethylene-diamine ligands, and/or dansyl fluorescence labels. The cytotoxicity was determined in vitro in concentration-dependent assays using the MCF-7 cell line. The internal structure of the presented dendrimers did not seem to play a profound role in cytotoxicity, despite the common view that the interior of low-generation dendrimers is accessible. The surface decoration, however, was crucial for toxic effects. Most of the examined non-charged dendrimers (e.g., the protected and dansylated ones) were non-toxic, although they were clearly bioavailable as proven by cell uptake experiments, and all completely diaminopropionic-acid-decorated dendrimers (positively charged) were also non-toxic. These results, especially the latter case, indicated that positive charges on a dendrimer surface did not always lead to cell toxicity and that another structure/toxicity correlation might also play a role [85]. 

Huang et al. also described synthesis of two non-peptidic fluorescently labeled Newkome-type dendrimers, differentiated over a varied alkyl spacer with guanidine end moieties. These novel nanomolecules were designed as biocompatible carriers of bioactive cargo able to cross the cell membrane and localize in targeted cell compartments. The behavior of dendrimers was comparable in two tested cell lines: NIH-3T3 fibroblasts and human microvascular endothelial cells (HMEC). The differential localization patterns of the two molecular transporters could be controlled through the variation of alkyl spacer length at the terminal generation of the dendrimer. Intracellular delivery of bioactive entities into specific subcellular locations utilizing this practical approach might overcome limitations in drug delivery [86].

## 4. Cytotoxicity of Dendrimers in the Aspect of Potential Clinical Applications

Although cytotoxicity is usually the limiting factor for dendrimer application, it plays a more comprehensive role in clinical practice. Dendritic polymers chosen for the potential treatment of infectious diseases should be able to kill a pathogen while remaining harmless for a patient. Similarly, in anti-cancer therapy, dendrimers should affect abnormal cells only. This effect can be achieved with proper dose selection and by utilizing specific ligands or effects such as “enhanced permeability or retention”. This section reviews recent research on the cytotoxicity of dendrimers in the context of their potential clinical applications.

PPI dendrimers were investigated for their cytotoxic and antibacterial activities by Felczak et al.; original PPI G4 dendrimers and PPI G4 with a surface modified by 25% and 100% maltose (PPI-25%mG4 and PPI-100%mG4, respectively) were evaluated for their antibacterial activity against Gram-positive bacteria, *Staphylococcus aureus* and *Staphylococcus epidermidis*, Gram-negative bacteria, *Escherichia coli* and *Pseudomonas aeruginosa*, and yeast, *Candida albicans*. Cytotoxicity of all tested dendrimers was checked on B14, human liver hepatocellular carcinoma (HepG2), N2a, and rat liver cell lines (BRL-3A). The obtained results indicated that unmodified PPI dendrimers and PPI dendrimers modified by 25% maltose displayed antibacterial activity and a striking selectivity toward *S. aureus*, at the concentration of 1 µM, which, at the same time, was harmless for the eukaryotic cell lines [31]. Klajnert et al. showed the properties of a series of new, low-molecular-mass, lysine-based peptide dendrimers with a varying distribution of cationic and aromatic groups in the structure. Lysine-based peptide dendrimers expressed antimicrobial activity against Gram-positive (*S. aureus)* and Gram-negative (*E. coli*) bacteria, as well as against a fungal pathogen (*C. albicans*). However, contrary to the PPI dendrimers, lysine-based peptide ones exhibited some level of toxicity, although most of them caused only a slight (ca. 20%) decrease in B-14 cell viability for minimum inhibitory concentrations. The study also showed that the degree of branching, steric distribution, and types of hydrophobic (aromatic) groups and cationic centers are important components of dendrimeric structure and influence both antimicrobial potency and toxicity [15]. 

Metallodendrimers (metal complexes of dendrimers) are another group of dendritic compounds. It was revealed that polyamide metallodendrimers possess anti-bacterial activity against *Bacillus subtilis* and *S. aureus* (Gram-positive), as well as *E. coli* and *Salmonella* typhi (Gram-negative). Metallodendrimers also showed anti-tumor activity against the MFC-cell line. Pt(II)-containing metallodendrimers were found to be more efficient in the induction of MFC-7 death than Pd(II)-containing metallodendrimers, and both showed lower cytotoxicity than cisplatin (standard drug) [87]. Pitto-Barry et al. showed that the entrapment of water-soluble dendrimer guests within metallaprism hosts led to apparent enhancements in cytotoxicity and preferential accumulation in tumors, making them interesting candidates for anticancer studies. Three generations of pyrenylbis-MPA dendrimers with two different end-groups, acetonide (pyrGn) or alcohol (pyrGn-OH) (*n* = 1–3), were synthesized, and the pyrenyl group of the dendritic molecules was encapsulated in the arene ruthenium metallacages. The host–guest systems inhibited the growth of both cisplatin-sensitive and -resistant cancer cells (A2780 and A2780cisR). Moreover, these water-soluble host–guest systems showed cytotoxicity toward the cisplatin-resistant cell line that was comparable to free cisplatin in non-resistant human ovarian cancer cells [88].

Thiagarajan et al. characterized the activity of a PAMAM dendrimer conjugated with an anticancer drug camptothecin (PAMAM–CPT) on human colorectal carcinoma cells (HCT-116). The conjugate was stable under physiological pH (7.4) in phosphate-buffered saline (PBS) and in growth media (with 10% FBS) with minimal release of 4% and 6% drug, respectively, at 48 h. PAMAM–CPT inhibited the proliferation of HCT-116 cells and induced cell-cycle arrest with up to 68% of cells blocked in the gap 2 (G2) phase. Thus, the PAMAM–CPT conjugate was active against colorectal cancer cells in vitro, inhibiting their growth and inducing nuclear fragmentation. However, it would be useful to test the cytotoxicity of this conjugate against normal cells [89]. 

Surface modifications of dendrimers were shown to be a factor enhancing the cell-penetrating ability of the drug–dendrimer complex, thus contributing to the inhibitory effect on cancer cell growth. Jia et al. modified PAMAM dendrimers G5 with acryloyloxyethyl phosphorylcholine (APC), a molecule possessing biomimetic properties. The hydrophobic interior of the modified dendrimer (G5-PC) was used to incorporate the anti-cancer drug adriamycin (ADR) and the G5-PC showed sustained release behavior for ADR. Cell viability and morphology analyses on the cancerous HepG2 cell line revealed that G5-PC was characterized by much lower toxicity in comparison to the unmodified PAMAM dendrimer. Furthermore, the drug-loaded G5-PC was shown to be internalized into cancer cells and could effectively decrease their viability [90]. Teow et al. also applied PAMAM G3 dendrimer as a drug carrier, increasing the permeability of paclitaxel (PTX), a poorly soluble anticancer drug. G3 dendrimers were surface-modified with lauryl chains and conjugated with PTX via a glutaric anhydride linker. Toxicity of the dendrimer and conjugates was tested using the human Caco-2 cell line and primary cultured porcine brain endothelial cells (PBECs). Cytotoxicity studies showed that the conjugation of lauryl chains and PTX on the G3 dendrimer significantly (*p* < 0.05) increased the cytotoxicity against both cell types. The conjugate had approximately 12-fold greater permeability across both apical and basolateral cell monolayers than that of PTX alone [91]. Paclitaxel was also conjugated with hydroxyl-terminated PAMAM G4 dendrimer and bis(PEG) polymer for the enhancement of drug solubility and cytotoxicity. Cytotoxicity of a PAMAM dendrimer–succinic acid–PTX conjugate toward A2780 human ovarian carcinoma cells was increased 10-fold as compared to free nonconjugated drug [92]. 

In another study, PEGylated PAMAM dendrimer of generation 3 was loaded with fluorouracil (5-FU) and tested in vitro on the MCF-7 cell line. The results indicated that the PAMAM dendrimer presented higher toxicity as compared to its PEGylated counterpart. However, PEG-modified PAMAM dendrimer loaded with 5-FU exhibited antiproliferative activity against the cancer cell line. An additional benefit from the encapsulation of fluorouracil in the PEGylated dendrimer was a slow release profile of the drug. Moreover, in vivo studies revealed that the tested complex exhibited a significant decrease in the volume of the tumors, which were generated by the MCF-7 cancer cells [93].

Despite their cytotoxicity, PPI dendrimers were also widely used as effective delivery vehicles for drugs. Wang et al. decreased the cytotoxicity of PPI dendrimers by acetylation and then used them to encapsulate anticancer drugs, including methotrexate sodium, sodium deoxycholate, and doxorubicin. The results indicated that the degree of acetylation determined the level of cytotoxicity and drug-loading capacity of the dendrimer. The drug-loading capacity of acetylated PPI dendrimers increased proportionally with the degree of acetylation on the dendrimer surface. PPI dendrimers with greater than 80% acetylation did not affect the viability of MCF-7 and A549 cells. The cytotoxicities of methotrexate sodium and doxorubicin were also significantly reduced when they were complexed with acetylated PPI dendrimers with high degrees of acetylation (>80%) owing to sustained drug release from the dendrimers. The results suggested that surface acetylation can reduce the cytotoxicity and improve the anticancer drug-loading capacity of cationic dendrimers [94]. Kesharwani et al. developed and compared the cancer-targeting potential of ligand-anchored folate-, dextran-, and galactose-anchored PPI dendrimers. Hemolytic studies demonstrated that free PTX was found to be maximally cytotoxic as compared with ligand-conjugated PPI dendrimers. The MTT assay showed that folate–PPI systems had maximum anticancer activity in all concentrations as compared with dextran- and galactose-based formulations. Kesharwani et al. determined the order of targeting efficiency of the three targeting ligands under investigation to be folate > dextran > galactose. These results confirmed that folate is the most efficient targeting ligand for targeting cancer cells when compared with dextran and galactose [95]. In order to increase delivery of paclitaxel across the blood–brain barrier (BBB), Patel et al. used thiamine-conjugated PPI dendrimers as drug carriers. PTX-loaded thiamine-conjugated PPI dendrimers (PTX–Tm-PPI) showed increased drug loading and reduced hemolytic toxicity with suitability for prolonged delivery of PTX during in vitro release. Ex vivo cytotoxicity studies of free PTX, PTX–PPI, and PTX–Tm-PPI dendrimers over the IMR-32 human neuroblastoma cell line revealed the higher potential of the PTX–Tm-PPI nanoconjugate to retard tumor cell viability as compared to plain PTX or PTX–PPI [96].

The use of vector molecules for the targeted delivery of antitumor drugs provides selectivity for cancer cells. Yabbarov et al. used a recombinant receptor-binding fragment of alpha-fetoprotein (rAFP3D) as a vector molecule. The receptor of alpha-fetoprotein, a tumor marker, is expressed on the surface of many tumor cells, but not in normal human tissues. The vector rAFP3D was conjugated with a PAMAM G2 dendrimer and an anticancer drug, doxorubicin (Dox). The conjugate demonstrated a high cytotoxicity against human ovarian adenocarcinoma cell lines (Dox-sensitive SKOV3 cells and Dox-resistant SKVLB cells), while having low toxicity against human peripheral blood lymphocytes [97]. Another possible strategy to improve targeting of anticancer drugs is to conjugate the dendrimer with biotin, which is considered as the most promising targeting molecule among vitamins [98,99].

Zhang et al. synthesized a saccharide-terminated PAMAM G3 dendrimer, which was then conjugated with the drug methotrexate (MTX). This construct was shown to have potential as an anticancer nanodevice for the specific targeting and killing of folate receptor (FR)-expressing tumor cells. Their results showed that G3–MTX presented enhancement in binding avidity to folate-binding protein (FBP) that was three orders of magnitude higher than a free folic acid (FA), and internalized into FR-expressing KB cells (a subline of the cervical carcinoma HeLa cells) in a dose-dependent and receptor-mediated fashion [100]. Han et al. found that major vault protein (MVP), similarly to P-glycoprotein (Pgp), might be involved in the drug resistance of human breast cancer MCF-7/ADR cells by transporting doxorubicin from the action target (i.e., nucleus) to the cytoplasm. In order to prevent this process, PAMAM dendrimers were functionalized with a polysaccharide hyaluronic acid (HA) to effectively deliver Dox, as well as MVP-targeted small interfering RNA (MVP-siRNA), to downregulate MVP expression and improve DOX chemotherapy in MCF-7/ADR cells. It was shown that DOX PAMAM–HA exhibited stronger cytotoxicity than Dox, and was characterized by better targetability, intracellular accumulation, increased blood circulating time, and less in vivo toxicity. Furthermore, co-delivery of siRNA and DOX by PAMAM–HA exhibited a satisfactory gene-silencing effect, as well as enhanced stability and efficient intracellular delivery of siRNA, which allowed Dox access to the nucleus and induced much stronger cytotoxicity than the siRNA-absent case as a result of MVP knockdown [101]. In another example, Han et al. conjugated a PEG-modified PAMAM dendrimer with the HAIYPRH (T7) peptide, a ligand specific for the transferrin receptor that is often overexpressed on cancer cells. The PAMAM–PEG–T7 construct was successfully loaded with Dox, formulating PAMAM–PEG–T7/DOX nanoparticles (NPs). The results indicated a significant enhancement of the cellular uptake of Dox. In vivo studies with T7-modified NPs also showed an increased accumulation of Dox in the tumor by approximately 1.7-fold compared to that of unmodified nanoparticles, and by approximately 5.3-fold compared to that of free Dox [102]. 

Gupta et al. added DOX (approximately 26 and 65%) to the PPI dendrimers, as well as folate-conjugated PPI (PPI–FA) dendrimers. PPI–FA–DOX exhibited the highest percentage cell-growth inhibition compared to other formulations and to the drug itself. All three formulations showed a dose-dependent inhibition of MCF-7 cells. The higher uptake in the case of PPI–FA–DOX was possibly due to the ligand-specific targeting of dendrimers due to surface conjugation of the folic acid [103]. 

The published toxicity levels of all dendrimers mentioned in this paper are summarized in Table 1.

## 5. Conclusions

Although there are active compounds that are able to inhibit or cure particular diseases, they are often not effective due to weak solubility, lack of ability to cross biological barriers, poor targetability, and sensitivity to the cell environment (pH) and its molecular machinery (enzymes, intracellular processing). A solution to this problem could be “packing” the active compound into or loading it onto a biocompatible delivery platform. One of the most promising options here is represented by dendrimers.

Dendrimers, which were synthesized in the 1980s, quickly attracted attention of researchers from the biomedical field, and, in the 1990s, first works concerning their biocompatibility, cytotoxicity, and potential applications appeared. Dendrimers were tested as carriers of various molecules and, simultaneously, their toxicity was examined using different cell lines. It was discovered that, in general, toxic effects caused by dendrimers were dependent on the generation, the number of surface groups, and the nature of terminal moieties (anionic, neutral, or cationic). A higher generation and an increased number of positive charges on the surface resulted in higher cytotoxicity. In order to decrease the cytotoxicity of dendrimers, scientists started to introduce different chemical modifications on the periphery of the nanomolecule. Dendrimers grafted with PEG, acetyl groups, carbohydrates, and other moieties did not affect cell viability, or did so only slightly, while still maintaining other advantageous properties. These properties include defined size, shape, and molecular weight, as well as monodispersity, the presence of the void space, tailorable structure, internalization by cells, selectivity toward cells and intracellular components, protection of guest molecules, and controllable release of the cargo. Intensive studies on dendrimers and their constant improvement resulted in their current status as systems of great potential in the biomedical field. In particular, they are developed as nanocarriers of drugs (anti-cancer, anti-infectious, anti-inflammatory, ophthalmic, and other), nucleic acids in gene therapy (DNA, anti-cancer, or anti-HIV siRNA), and imaging agents for MRI (e.g., gadomer, Gd-DTPA). Furthermore, some dendrimers also exhibit intrinsic biological activity, with anti-bacterial, anti-fungal, and anti-viral properties of dendritic nanomolecules previously demonstrated. Moreover, other dendrimers were reported to inhibit processes involved in neurodegenerative disorders, i.e., fibrillation and aggregation of specific proteins. Lastly, in several cases, it was demonstrated that dendrimers can be toxic to cancerous cells, while exhibiting no or low toxic effects toward normal cells. A synergistic effect of the combination of the active dendrimer and its cargo molecules (drugs) can result in a very potent therapy.

The unique structure of dendrimers enables modifying the surface groups, which are largely responsible for properties of dendrimers, including cytotoxicity. However, it should be taken into account that diminishing cationic charge on the surface often reduces the therapeutic activity of dendrimers, e.g., the ability to create complexes with DNA or RNA. Therefore, partial modification is sometimes applied in order to achieve a balance between reduced toxicity and retained activity; this is frequently done via partial modification of PPI dendrimers by maltose or maltotriose [104].

Depending on the specific medical application, a higher or lower dose of the dendrimer is needed. It allows finding the right dendrimer for a specific purpose from the large library of available dendrimers. Therefore, the toxicity of the dendrimers is not the main issue that slows down the process of commercializing dendrimers. However, this field certainly needs better consistency when testing the long-existing and new compounds. Table 1 clearly demonstrates that it is difficult to compare the toxicity of different types of dendrimers, since the experimental approaches applied in cytotoxicity studies differ substantially.

Overall, the intrinsic cytotoxicity of dendrimers can be overcome by precise chemical modifications. Their complexation with various bioactive molecules can be perfectly optimized, while being delivery-specific and safe for both the cell and the cargo. At present, dendrimers are an outstanding alternative to other drug carrier platforms by increasing the bioavailability and efficiency of transported compounds, thereby enhancing their therapeutic effects.

## Figures and Tables

**Table 1:** Levels of cytotoxicity of chosen dendrimers. The most promising are lysine-based dendrimers, polyester dendrimers, and poly(amido amine) (PAMAM) dendrimers due to their non-toxicity or extremely high half maximal inhibitory concentration (IC_50_) levels. The table also illustrates the generation (G) dependency of cytotoxicity of dendrimers and the positive effects of a few particles on the cytotoxicity when they are conjugated to dendrimers (i.e., maltose, maltotriose on poly(propylene imine) (PPI); pyrrolidone, lauroyl on PAMAM).

Dendrimer	Cell Line	Level of Cytotoxicity IC_50_	Reference
lysine-based peptide dendrimers R 121 R 131 R 132 R 124 R 155 R 169	B14 (Chinese hamster fibroblasts)	1590 μM 1070 μM 630 μM 580 μM 490 μM 280 μM	Klajnert et al. [15]
PPI-G4 PPI-G4–25% maltose PPI-G4–100% maltose	B14 (Chinese hamster fibroblasts) N2a (mouse neuroblastoma) BRL-3A (rat liver derived cells) HepG2 (human liver hepatocellular carcinoma)	PPI G4 3.18 µM 3.34 µM 5.93 µM 6.91 µM PPI-m25%/PPI-m100% IC_50_ > 100 µM	Felczak et al. [31]
PAMAM dendrimers G4PEG2 G4PEG4	Caco-2 (colon adenocarcinoma)	PEG2/PEG4 (μM) 120/790	Jevprasesphant et al. [49]
PAMAM generations 1.5–3.5) DAB (generations 1.5–3.5) PAMAM (generations 1–2) PAMAM (generations 3–4) DAB (generations 2/3/4) PEA (generations 1–2) PEA (generations 3) CSi-PEO (generations 1–2)	B16F10 (murine melanoma cells)	IC_50_ >155 µM IC_50_ >570 µM IC_50_ > 614 µM 35 µM 178/14.5/7.2 µM IC_50_ > 60 µM 14.3 µM IC_50_ > 16.2 µM	Malik et al. [50]
PAMAM–pyrrolidone dendrimer	N2a (mouse neuroblastoma)	<200 μM non-toxic	Ciolkowski et al. [51]
PAMAM dendrimers generation 4 (G4) generation 5 (G5) generation 6 (G6)	HaCaT (human epidermal keratinocytes) SW480 (primary adenocarcinoma of colon)	G4/G5/G6 (μM) 3.21/1.07/1.02 ♦ 16.35/1.89/1.30 ♣ 23.16/5.75/3.17 ✚ 1.44/0.37/1.16 ♣ 8.3/3.12/1.56 ♣ 10.8/4.33/1.87 ✚	Mukherjee et al. [53]
PAMAM dendrimers generation 4 (G4) generation 5 (G5) generation 6 (G6)	PLHC-1 (fish hepatoma cel line) RTG-2 (rainbow trout gonad tissue) vs. *Daphnia magna*	G4/G5/G6 (µM) 2.08/12.93/0.68 * 0.56/6.07/0.27 * 0.21/2.51/0.13 *	Naha et al. [56]
PAMAM–pyrrolidone dendrimer	B14 (Chinese hamster fibroblasts) mHippoE-18 (mouse embryonic hippocampal cells) BRL-3A (rat liver derived cells)	Non-toxic	Janaszewska et al. [59]
carboxymethylchitosan/poly(amidoamine) (CMCht/PAMAM) dendrimer	U87MG (glioblastoma GBM) hTERT/E6/E7 (human immortalized astrocytes)	Non-toxic	Pojo et al. [61]
PAMAM G5 core–PAMAM G2.5 shell tecto-dendrimers	HaCaT (human epidermal keratinocytes) Caco-2 (colon adenocarcinoma) SK-Mel-28 (human melanoma)	up to 50 μM non-toxic up to 50 μM non-toxic IC_50_ = 7.5 μM	Schilrreff et al. [62]
Amino-terminated PAMAM dendrimers G3 and G4 Amino-terminated PAMAM dendrimers G3 and G4 with N-(2-hydroxydodecyl) groups	H4IIE (rat hepatoma)	G3/G4 (μg/mL) <500 non-toxic 12.96/38.3	Hernando et al. [63]
Histidine-activated PAMAM generation 4 (HPG4) PAMAM G4 HPG4/pDNA	MDA-MB-231 (breast cancer)	0.15 mg/mL 0.06 mg/mL cell viability > 80%	Wen et al. [64]
PAMAM dendrimers (L for lauroyl) G2 G2L6 G2L9 G3 G3L6 G3L9 G3L13 G4 G4L3 G4L6 G4L9 G4L15 G2.5 G3.5	Caco-2 (colon adenocarcinoma)	IC_50_ >1000μM IC_50_ >15000 μM 1060 μM 1400 μM IC_50_ > 1000 μM 310 μM 220 μM 130 μM 360 μM IC_50_ > 1000 μM 100 μM 40 μM IC_50_ > 1000 μM IC_50_ > 1000 μM	Jevprasesphant et al. [65]
PAMAM dendrimer G3.5 PAMAM dendrimer G4 PPI-G4 PPI-G4–25% maltotriose PPI-G4–100% maltotriose	SKOV3 (human ovarian carcinoma) CHO (Chinese hamster ovary)	SKOV3/CHO IC_50_ > 300 µM/IC_50_ > 300 µM 5.56 µM/46.49 µM 7.79 µM/14.70 µM IC_50_ > 300 µM/100 µM IC_50_ > 300 µM/144.60 µM	Janaszewska et al. [66]
PPI-5.0G DBG t-BOC (protected glycine, *t*-butyl) DBPA t-BOC (protected phenylalanine, *t*-butyl) M-PPI (mannose) L-PPI (lactose)	HepG2 (human liver hepatocellular carcinoma) COS-7 (African green monkey kidney)	HepG2/COS-7 0.58 mg/mL/0.62 mg/mL 5.53 mg/mL/5.74 mg/mL 6.15 mg/mL/7.21 mg/mL 2.35 mg/mL/2.46 mg/mL 6.19 mg/mL/5.89 mg/mL	Agashe et al. [67]
PAMAM dendrimers generation 2 (G2) generation 4 (G4) generation 6 (G6) lipidated generation 4 (G4C12)	HeLa (human cervical cancer) PC-12 (neuronal-like) HepG2 (human hepatocarcinoma) MRC5 (human lung fibroblast) primary astrocytes	Non-toxic 1.5 μM for 1 h	Albertazzi et al. [68]
heterobifunctional G4-PAMAM G4-PAMAM-NH2 G4-PAMAM-OH G4-PAMAM-NH-Ser(OH)-NH2 G4-PAMAM-NH-Cys(SH)-NH2 G4-PAMAM-OH-Cys(SH)-NH2	A549 (human lung adenocarcinoma)	n.d.	Navath et al. [72]
CPD-G2 CPD-G3	mHippoE-18 (mouse embryonic hippocampal cells) N2a (mouse neuroblastoma)	mHippoE-18/N2a 2.33 µM/1.84 µM 1.31 µM/1.74 µM	Lazniewska et al. [76]
CPD G3 CPD G4	human lymphocytes	G3/G4 (15 µM) 5.15/4.00	Gomulak et al. [77]
copper(II)-conjugated phosphorus dendrimers	HCT116 (human colon cancer) MCF7 (hormone-responsive breast cancer) OVCAR8 (ovarian carcinoma) U87 (human glioblastoma–astrocytoma, epithelial-like) MCR5 (proliferative human lung fibroblasts) EPC (endothelial progenitor cells)	Increased with the number of terminal moieties	El Brahmi et. al. [79]
polyester-based nanocarriers	MDAMB468, MDA-MB23, MCF7 (human breast carcinoma) A498 (human kidney carcinoma) Raw 264.7 (mouse macrophage)	<300 μg/mL Non-toxic	Zeng et al. [80]
hydroxylated polyester dendrimers G1–G5 used as stabilizing agent of silver particles	A549 (human lung adenocarcinoma)	Non-toxic	Mahltig et al. [81]
bis-MPA polyester dendrimers	HeLa (human cervical cancer) THP.1 (acute monocytic leukemia) primary human monocyte	Non-toxic	Feliu et al. [82]
metallodendrimers PtG1 PdG1 free cisplatin	MFC-7 (human breast adenocarcinoma)	30.0 µM 50.0 µM 2.33 µM	Ahamad et al. [87]
Pyrenyl-containing dendrimers pyrG1-OH pyrG2-OH pyrG3-OH host–guest systems [pyrG1-OH⊂2][CF_3_SO_3_]_6_ [pyrG2-OH⊂2][CF_3_SO_3_]_6_ [pyrG3-OH⊂2][CF_3_SO_3_]_6_ free cisplatin	A2780 (human ovarian cancer) A2780cisR (human ovarian cancer cisplatin resistant);	A2780/A2780cisR n.d./n.d. 5.9 μM/7.4 μM 8.7 μM/8.7 μM 0.8 μM/2.2 μM 4.1 μM/1.9 μM 1.5 μM/1.5 μM 1.6 μM/8.6 μM	Pitto-Barry et al. [88]

◆—MTT assay; ♣—alamarBlue assay; ^✚^—Neutral Red assay; *—ecotoxicological assay; n.d.—no data.
